# The occurrence and molecular identification of *Thelazia* spp. in European bison (*Bison bonasus*) in the Bieszczady Mountains

**DOI:** 10.1038/s41598-022-27191-x

**Published:** 2022-12-29

**Authors:** Katarzyna Filip-Hutsch, Zdzisław Laskowski, Anna W. Myczka, Michał Czopowicz, Bożena Moskwa, Aleksander W. Demiaszkiewicz

**Affiliations:** 1 Department of Food Hygiene and Public Health Protection, Institute of Veterinary Medicine, Warsaw University of Life Sciences–SGGW, Nowoursynowska 159, 02-776 Warsaw, Poland; 2 Witold Stefański Institute of Parasitology, Polish Academy of Sciences, Twarda 51/55, 00-818 Warsaw, Poland; 3 Division of Veterinary Epidemiology and Economics, Institute of Veterinary Medicine, Warsaw University of Life Sciences–SGGW, Nowoursynowska 159C, 02-776 Warsaw, Poland

**Keywords:** Parasitic infection, Ecology, Zoology

## Abstract

Infection with *Thelazia* nematodes results in eye disease in wild and domestic animals. The aim of the present study was to describe the occurrence of *Thelazia* nematodes in European bison, and to subject the isolated parasites to molecular identification and phylogenetical analysis. The eyeballs of 18 European bison from the Bieszczady Mountains, culled due to dysfunctional vision, were collected for study. The conjunctival sacs, tear ducts, corneal surface and nictitating membrane were rinsed with a saline solution. Any obtained nematodes were isolated under a stereoscopic microscope, and then identified as *T. gulosa* or *T. skrjabini* by molecular analysis of partial *cox1* sequences. The prevalence of infection with *Thelazia* spp. was found to be 61%, with a 95% confidence interval (CI 95%) of 39–80%. *Thelazia skrjabini* was isolated from 56% (CI 95% 34–75%) of examined animals; *T. gulosa* was significantly less common (p = 0.038) with the prevalence of infection reaching 22% (CI 95% 9–45%). Three European bison were cross-infected with both *T. gulosa* and *T. skrjabini*. Phylogenetic analysis found the obtained sequences to be similar to those of *Thelazia* species from domestic ungulates in Europe. Infection intensity ranged from 1 to 16 nematodes per individual (median of three nematodes), and was significantly higher in females (6 nematodes) than in males (1 nematode; p = 0.019). A tendency for seasonal occurrence of nematodes in European bison was also observed. Our study provides further information regarding the patterns of *Thelazia* transmission in European bison in Poland.

## Introduction

Infectious diseases contribute to a species being classified as endangered^1^ and can cause significant temporary or permanent declines in local populations^2^. Parasitic diseases in particular may have a substantial impact on wildlife population dynamics and represent a critical issue in the conservation of endangered species^3^. The recent changes in the climate appear to support parasite development and survival rates, as well as disease transmission and host susceptibility^4,5^; as such, the emergence of new parasitic diseases, and the re-emergence of previous ones, may soon represent a major threat to wildlife diversity. One recent example of a disease that existed previously in domestic ruminants but has recently begun to rapidly spread in wildlife is thelaziosis, which has been noted in populations of endangered European bison (*Bison bonasus*) in Poland, a middle European country^6^.

Nematodes of the genus *Thelazia* are known to cause eye diseases in wild and domestic animals worldwide^7^. Several *Thelazia* species specific to particular hosts have been identified in Europe, and ruminants may be exposed to *T. gulosa*, *T. skrjabini*, *T. rhodesi* and *T. lacrymalis*^8^. *Thelazia gulosa* is also considered a parasite of zoonotic potential and has recently been identified in humans in the United States of America^9^. *Thelazia lacrymalis* and *T. rhodesi* have also been isolated from the eyeballs of a horse^7,10^.

Adult nematodes localize in the conjunctival sac and tear ducts, under the nictitating membrane and on the cornea of infected animals^6,7^. The eye worms are transmitted by secretophagous non-biting flies from the genus *Musca*, which become infected with the first stage larvae while feeding on animal lacrimal secretions^6,11^. In the intermediate host, the first-stage larvae develop into the invasive third stage, migrate to the fly suckers and are passed to the conjunctival sac of the next definitive host^11^. The pathogenic effect of *Thelazia* nematodes derives from the mechanical irritation of the conjunctiva and cornea, as well as the toxic effect of parasitic metabolites. The infected animals suffer from acute conjunctivitis, often complicated by secondary bacterial infections leading to purulent eye inflammation, corneal opacity, and ulceration^6,8^.

In Poland, nematodes of the species *T. gulosa* and *T. skrjabini* were commonly detected in cattle in the 1960s and 1970s, especially in densely-populated herds^12–14^. Infection manifested itself only as transient blindness, probably due to regular deworming of livestock, and was of little concern^6^. However, the incidence of thelaziosis in European bison has been increasing in Poland since 2018. The animals suffer from lesions of the eyeballs, leading to visual impairment, blindness, and eventually death^6^. As such, thelaziosis is becoming a matter of veterinary concern in wild ruminants in Poland and warrants regular monitoring.

European bison is an endangered species that went extinct in the wild at the beginning of the 1900s; the population has been restored from captive individuals since the 1950s and eventually reintroduced to European forests^15^. Currently, over 7000 free-living European bison can be found worldwide, of which about 2300 (one-third of the world population) live in Poland. One of the largest populations in the country, comprising over 600 free-living individuals, inhabits the Polish part of the Bieszczady Mountains; the population itself is divided into eastern and western subpopulations^16^.

Currently, the European bison population is managed with the aim of preserving its genetic diversity and establishing new herds, which involves relocating free-living and captive animals between European countries^17^. As international transport of animals might favour the spread of some pathogens to new areas^18^, there is a particular need to monitor the occurrence of *Thelazia* species in European bison in Poland.

The aim of our study was to determine the prevalence and the species diversity of nematodes of the genus *Thelazia* in the free-living population of European bison in the Bieszczady Mountains in Poland, and to subject isolated parasites to molecular identification and phylogenetic analysis.

## Results

### Study population

The study population comprised 18 bison, nine males and eight females, aged from 6 to 18 years (median 14 years) (sex and age unknown for 1 bison). The median age of the males and females was 15 and 11 years, respectively, and did not differ significantly between sexes (p = 0.068). Males were significantly heavier (median 710 kg, range 350–800 kg) than females (median 400 kg, range 350–450 kg) (p = 0.009) (body weight unknown for 2 bison).

### Anatomopathological analysis

The most common lesions were bilateral conjunctival congestion together with fibrin deposits and corneal opacity. Corneal ulceration, purulent ocular inflammation and atrophic eyeballs were less common. Changes led to vision disfunction or blindness in the animals, which resulted in their culling.

### Indices of *Thelazia* infection

The overall prevalence of nematodes of the genus *Thelazia* was 61% (95% confidence interval (CI 95%) 39–80%; 11/18 bison) with the prevalence of *T. skrjabini* being significantly higher than of *T. gulosa* (p = 0.038) (Table 1). Co-infection with both *Thelazia* species was observed in three examined European bison, whereas seven were infected only with *T. skrjabini* and one was infected only with *T. gulosa*.

**Table 1:** Prevalence (percentage of infected bison) and intensity of infection (number of parasites in a single bison) with nematodes of the genus *Thelazia* in European bison in the Bieszczady Mountains.

Parasite	Overall			Right eye		Left eye	
	Number of infected bison	Prevalence (CI 95%)	Intensity of infection	Number of infected bison	Intensity of infection	Number of infected bison	Intensity of infection
*Thelazia* spp.	11	61 (39–80)	3 (1–16)^a^	9	2 (1–7)^a^	8	3 (1–9)^a^
*T. skrjabini*	10	56 (34–75)	2 (1–16)^a^	8	1 (1–7)^a^	7	3 (1–9)^a^
*T. gulosa*	4	22 (9–45)	2 (1–3)^a^	2	2, 3	3	1, 1, 1

^a^Median and range.

The number of nematodes detected in a single bison (i.e. the intensity of infection) ranged from 1 to 16, and this value did not differ significantly between animals infected with one *Thelazia* species and those with mixed infection (p = 0.375) (Table 1). The intensity of infection did not differ significantly between the left and right eyes of an infected bison (p = 0.859) and was not correlated with age (Spearman’s rank correlation coefficient (R_s_) =  − 0.18, p = 0.628) nor body weight (R_s_ =  − 0.55, p = 0.098). The intensity of *Thelazia* infection was significantly higher in females than males (p = 0.019). The highest intensity of *Thelazia* spp. infection was observed in August and the lowest in May; however, no statistical analysis was feasible due to the low number of examined animals (Supplementary Fig. S1).

### Molecular and phylogenetic analysis

The molecular analysis of the partial *cox*1 gen amplified from the isolated nematodes yielded two species: *T. gulosa* (OL362019) and *T. skrjabini* (OL362009).

**Figure 1:**
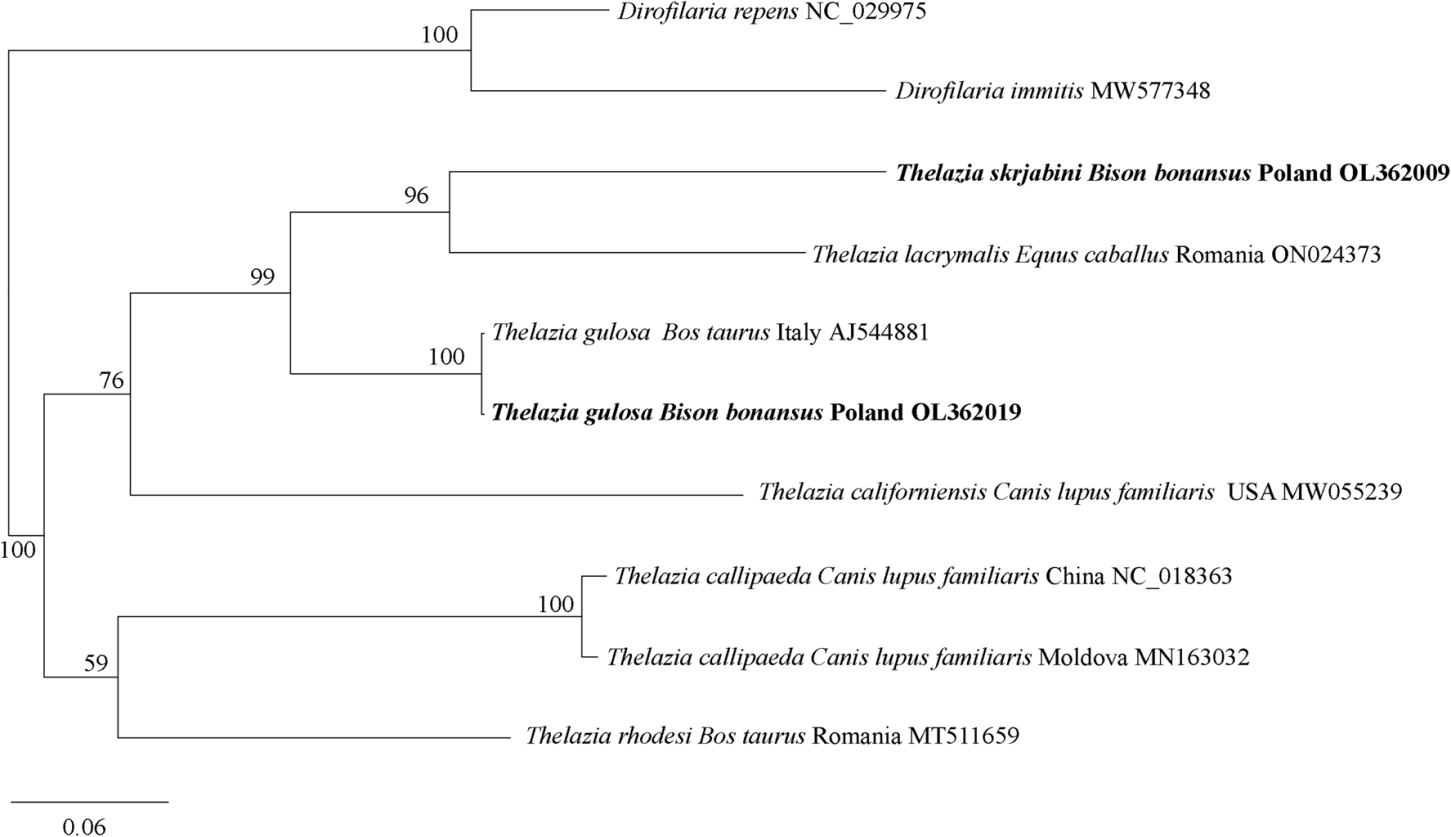
Phylogenetic tree of *Thelazia* spp. based on *cox1* partial sequences, constructed by Bayesian inference (BI) analysis using MrBayes version 3.2. The GTR+I+G model was chosen as the best-fitting nucleotide substitution model using JModelTest version 2.1.10 software^45,46^. Analysis was run for 1,000,000 generations, with 250,000 generations discarded as ‘burn-in’. Nodal support is indicated as Bayesian posterior probabilities. Sequences of *Dirofilaria repens* and *D. immitis* were used as the outgroup. Sequences from this study are in bold. The scale bars are proportional to the number of substitutions per site.

The sequence of *T. gulosa* obtained from European bison was identical to that isolated from bovines in Italy (AJ544881). However, the obtained sequence of *T. skrjabini* did not demonstrate a 100% similarity with any submission in the GenBank library. Our obtained sequence is the first molecular record of the *cox1* gene from *T. skrjabini*. Phylogenetic analysis of the *cox*1 sequences (Fig. 1), with *Dirofilaria repens* and *D. immitis* as an outgroup, revealed the presence of two clades. Both sequences obtained during the present study were included in the same clade, together with *T. californiensis* from a dog in the USA (MW055239), but in different subclades. *Thelazia gulosa* was grouped together with the isolate from the bovine in Italy (AJ544881) and *T. skrjabini* with *T. lacrymalis* from a horse in Romania (ON024373). The second clade included *T. rhodesi* isolated from cattle in Romania (MT511659) and sequences of *T. callipaeda* from a dog in Moldova (MN163032) and China (NC_018363).

## Discussion

Being highly exposed to the fly vectors of eyeworms, free-grazing animals such as bovids are considered especially vulnerable to *Thelazia* spp. infection^8^. As such, bovine thelaziosis, caused by nematodes of the species *T. gulosa*, *T. rhodesi*, and *T. skrjabini*, is the most commonly-reported animal thelaziosis^19^. Many studies concerning the occurrence of *Thelazia* nematodes in domestic cattle have been published in Europe, including the United Kingdom, Italy, Romania, Denmark, Ukraine, and Poland^12,13,19–24^; however, the reports of thelaziosis in wild ruminants are more limited. During the last century, a few asymptomatic *Thelazia* infections have been observed in European bison in Poland^25,26^. Since 2018, the number of clinical cases of thelaziosis has been begun to surge^6^. Not surprisingly, a high number of individuals were found to be infected with *Thelazia* spp. nematodes in our present study (60%), and all examined animals suffered from vision disfunction. It has been proposed that the disappearance of adult nematodes in eyes may resemble the self-cure phenomenon of gastrointestinal *Strongyles* in sheep^21^; however, little data is available to support this hypothesis.

Two species of *Thelazia* nematodes were isolated from the eyeballs of European bison in the Bieszczady Mountains, viz*. T. skrjabini* and *T. gulosa*, both considered typical of ruminants^8^. Although *T. skrjabini* has rarely been reported as a cause of bovine thelaziosis in Europe^19^, it was found to be significantly more prevalent in the studied European bison than *T. gulosa*.

Three European bison were cross-infected with both *T. gulosa* and *T. skrjabini*; this has previously been observed by other authors and is believed to be a consequence of the sympatric occurrence of the species in the environment^27^. Cross-infections might be also related to high host exposure and susceptibility to the parasite^28^; however the number of isolated *Thelazia* nematodes did not differ significantly between European bison with single-species and mixed infection.

*Thelazia gulosa* and *T. skrjabini* were placed in the same clade, as sister taxa, together with *Thelazia* nematodes from domestic ungulates in Europe (Fig. 1). This might indicate that domestic hosts act as possible sources of *Thelazia* infection for European bison in the Bieszczady Mountains^6,29^. The close phylogenetic relationship of European bison with domesticated bovids might increase the risk of cross‐species transmission of some parasites^3^, and possibly *Thelazia* nematodes. Currently, no data is available regarding bovine thelaziosis in Poland as it has not been monitored in cattle herds for the previous 50 years^12–14^. Thompson^30^ proposes that the lack of such monitoring programs in domestic hosts might account for an increased exposure of wildlife to parasites of domestic livestock. As such, it is possible that cattle may play a role in the transmission of *Thelazia* nematodes to free-living European bison.

*Thelazia rhodesi*, a common etiological factor of bovine thelaziosis^8,19,31^, was not found in any European bison in the present study. Indeed, phylogenetical analysis revealed that *Thelazia* nematodes isolated from the studied European bison were genetically distinct from *T. rhodesi*. The occurrence of *T. rhodesi* is expected to be restricted to countries with warmer climates than Poland, especially those in the southern part of Europe, such as Italy and Romania^19,24^.

Among the studied European bison, the females demonstrated significantly higher *Thelazia* nematode infection intensity than the males. Lower parasitic loads have also been observed in bison bulls by other authors, and it has been proposed that this difference probably results from the solitary lifestyle of the males: the females tend to live in groups, together with calves and subadults, which favours the spread of parasitic diseases^4,32,33^. In addition, females might be more susceptible to the infection due to immunosuppression caused by pregnancy or lactation^34^. In addition to reproductive status, host immunity may also vary in relation to stress or food availability^35^. It has been proposed that the nutritive status of the animals might influence their resistance to parasitosis^36^; indeed, studies indicate that greater food consumption favoured the immunocompetence of females and resulted in lower female-biased parasite infestation^37^. However, as several different mechanisms are responsible for gender-biased parasitism^37^, it is difficult to identify specific factors affecting higher *Thelazia* infection intensity in female European bison.

Eyeworm transmission requires the continuous presence of vectors in the environment^38^, and the occurrence of nematodes demonstrates seasonal variation dependent on the activity of the intermediate hosts^6,39^. Although the small number of examined animals in our study was too low to perform statistical analysis of seasonal variation, a higher intensity of *Thelazia* infection was observed in the summer compared to spring and autumn (Supplementary Fig. S1). Thus, the increasing transmission and infectivity of *Thelazia* spp. in European bison might be associated with climate variability and higher fly activity.

Two species of the genus *Musca*, viz*. M. domestica* and *M. autumnalis*, have so far been detected in the Bieszczady Mountains^40,41^, although no current data on their distribution is available. Both flies were also observed on large livestock farms in the adjacent region of Southern Poland^42^. It has been proposed that the widespread occurrence of *M. domestica* and *M. autumnalis* in the natural and semi-natural environment may result from them being synanthropic fly species connected with livestock husbandry^40^. Although it has been suggested that *M. domestica* may play a role in *Thelazia* transmission^8^, *M. autumnalis* is considered the main vector^43^ and might participate in passing the infection between sylvatic and synanthropic ruminant species. As there are no current data of *Thelazia* occurrence in other wild and domestic ruminants in Poland, it is important to begin regular eyeworm monitoring in areas known to be at risk of thelaziosis in European bison.

*Thelazia* spp. infections in wild ruminants in Europe are probably more widespread than previously assumed, and further studies are needed to identify the patterns of *Thelazia* transmission; this is particularly important given a zoonotic potential of the infection. Understanding the epidemiology of *Thelazia* spp. in European bison is essential for reducing the risk of infection, for example, by improving management strategies to avoid exposure to worms through the eyes. Future studies are needed to determine the occurrence of nematodes in the domestic cattle herds in Poland and to identify the role of *Muscidae* flies as vectors of bovine thelaziosis.

## Materials and methods

### Study area

**Figure 2:**
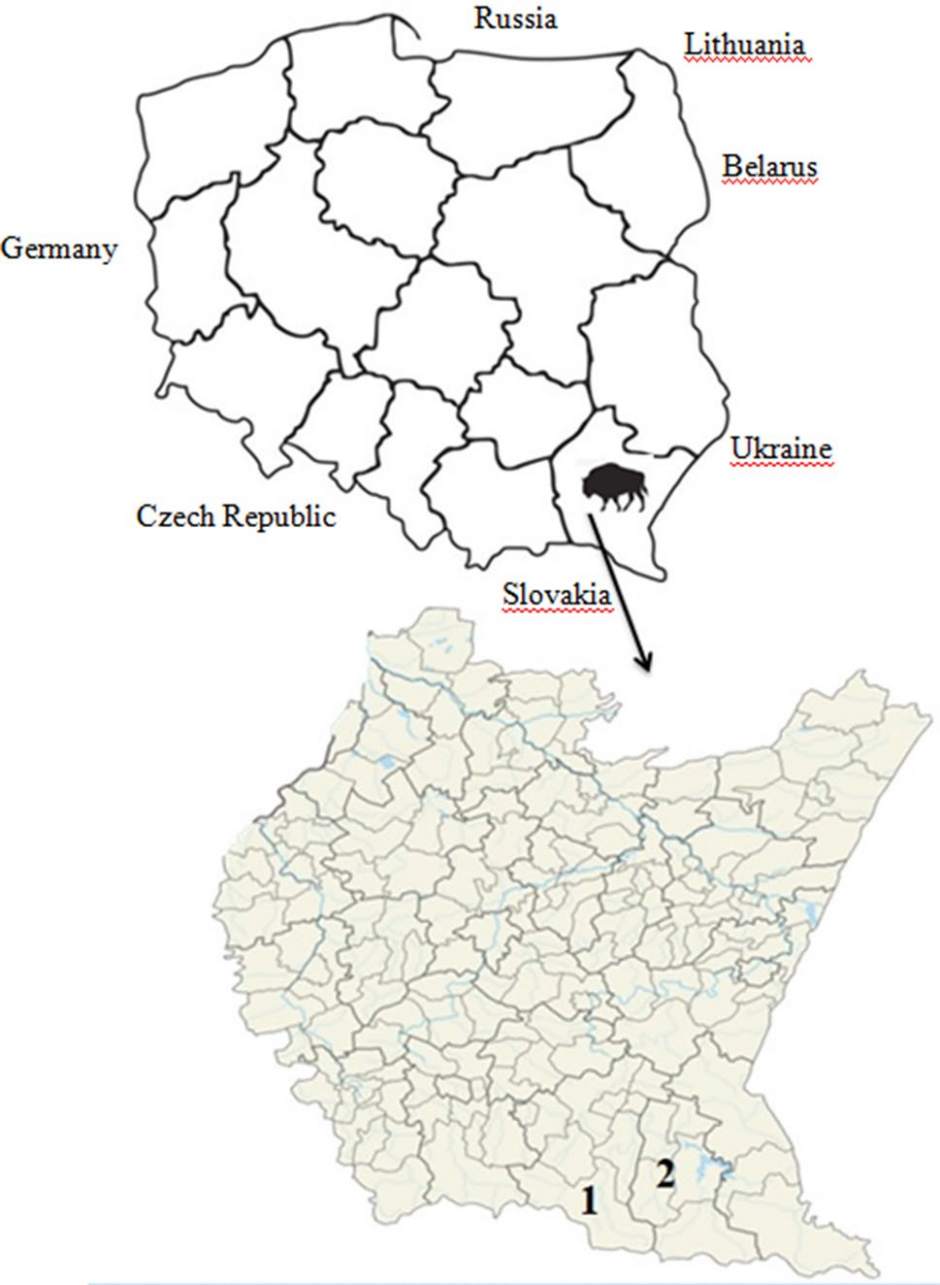
Study area showing the locations of the Forest Districts: Komańcza (1) and Baligród (2) (Microsoft Paint 11.2208.6.0; www.microsoft.com).

The material was collected in the Baligród and Komańcza Forest Districts, in the Polish part of the Bieszczady Mountains, south-eastern Poland (Fig. 2). The area is protected by the Cisna-Wetlina Landscape Park and The East Carpathian Biosphere Reserve. The landscape of the Bieszczady Mountains is characterized by parallel, long mountain ranges running from the north-west to the south-east, with the height gradually increasing from west to east. The highest peak of this part of the Bieszczady Mountains is Tarnica (1346 m above sea level). The climate is temperate and warm, and predominantly continental. The mean annual temperature is 7.5 °C and the annual precipitation ranges from 800 to 1200 mm, with the highest rates in the summer and the lowest during winter. The slopes and valleys are covered by Carpathian beechwood complexes, with an admixture of sycamore and fir trees, and the streams are lined by alder forests. Beechwood forests grow up to 1150 m above sea level and lie directly alongside the mountain pastures^44^. Agriculture is dominated by breeding and grazing of sheep and cattle. Agricultural lands mostly consist of meadows and pastures.

### Material collection and parasitological examination

Between May and October of 2019 and 2020 18 free-living European bison from the western subpopulation in the Baligród and Komańcza Forest Districts in the Bieszczady Mountains were culled due to apparent lesions on the surface of eyeballs. During the postmortem examination, age was estimated based on tooth development and wear, and the sex and body weight of the animals were recorded. The eyeballs were collected, together with adjacent tissues, transported at 4 °C to the laboratory, and examined.

In the laboratory, the anatomopathological changes of the cornea and conjunctival sacs were subjected to macroscopic examination. Then, the conjunctival sacs, tear ducts, corneal surface and the nictitating membrane were rinsed with physiological solution. The eyeballs were dissected and their structures thoroughly rinsed. The decantated sediment was examined for the presence of nematodes under a stereoscopic microscope (Polskie Zakłady Optyczne, Poland) at × 40 magnification. Any obtained parasites were isolated and identified to the species level based on morphometrical features^6^. The nematodes were subsequently preserved in 70% alcohol and stored for further molecular analysis.

### Molecular and phylogenetic procedures

To disrupt the surface of the nematodes, they were cut in half and mechanically ground with sterile sand (50 mg). DNA was isolated from nematode specimens with a commercial DNA Mini Kit (Syngen, Poland) according to the manufacturer’s protocol, with some modifications.

The DNA was amplified by PCR using primers designed in the Witold Stefański Institute of Parasitology, Polish Academy of Sciences: COIintF (5′-TGATTGGTGGTTTTGGTAA-3′) and COIintR (5′-ATAAGTACGAGTATCAATATC-3′). As a result, a 640 bp fragment of the mitochondrial cytochrome c oxidase subunit 1 gene (*cox1*) was obtained. The reactions were conducted in a 40 µl reaction mixture containing 2.0 µl of DNA template, 0.2 µl (1U) of Color Taq DNA Polymerase (EURx, Poland), 1 µl of dNTPs mix (10 mM), 0.5 µl of each primer (20 mM), 5 µl of 10× Polymerase buffer (pH 8.6, 25 mM MgCl2), and 30.8 µl of H_2_O. Nuclease-free water was added to the PCR mixture as a negative control. DNA amplification was performed using the DNA Engine T100 Thermal Cycler (BioRad, USA) according to the following program: denaturation at 94 °C for one minute, followed by 34 cycles of denaturation at 95 °C for 20 s, annealing at 56 °C for 20 s and extension at 72 °C for 40 s, with a final extension performed at 72 °C for 5 min.

The PCR products were visualized on a 1.2% agarose gel (Promega, USA) stained with SimplySafe (EURx, Poland) and a size-marked DNA Marker 100 bp LOAD DNA ladder (Syngen, Poland). Visualization was performed using ChemiDoc, MP Lab software (Imagine, BioRad, USA). The obtained PCR products were purified with the DNA clean-up Kit (Syngen, Poland). Following purification, the products were sequenced by a commercial facility (Genomed, Poland) and assembled using ContigExpress, Vector NTI Advance v.11.0 (Invitrogen Life Technologies, New York, NY, USA).

The obtained sequences were compared with those from GenBank in BLAST (NCBI, USA) and submitted to GenBank. A phylogenetic tree of *Thelazia* spp. based on *cox1* partial sequences was constructed by Bayesian inference (BI) analysis using MrBayes version 3.2. The GTR+I+G model was chosen as the best-fitting nucleotide substitution model using JModelTest version 2.1.10 software^45,46^. The analysis was run for 1,000,000 generations, with 250,000 generations discarded as burn-in. The phylogenetic trees were visualized using the TreeView software (S&N Genealogy Supplies, UK).

### Statistical analysis

Categorical variables were presented as counts and percentages, and compared between groups using the maximum likelihood G test, or Fisher’s exact test if the expected count in any cell of the contingency table was below 5. The 95% confidence intervals (CI 95%) for proportions were calculated using the Wilson’s score method^47^. Numerical variables were presented as the median and range, and compared between groups using the Mann–Whitney U test (unpaired groups) or the Wilcoxon’s signed rank test (paired groups). Correlations between two numerical variables were determined using the Spearman’s rank correlation coefficient (R_s_). The significance level (α) was set at 0.05. Statistical analysis was performed in TIBCO Statistica 13.3 (TIBCO Software Inc., Palo Alto, CA).

### Ethics declaration

The authors confirm that the ethical policies of the journal, as noted on the journal’s author guidelines page, have been adhered with accordance to Directive 2010/63/EU of The European Parliament and of The Council of 22 September 2010 on the protection of animals used for scientific purposes. All European bison were legally culled on the basis of permission from the General Directorate for Environmental Protection in Poland. The decision of General Directorate for Environmental Protection was implemented by the Regional Head Office of National Forests in Krosno, which supervised animals sacrifice. European bison as wild animals, were culled by professional hunters with respect to the animals welfare and safety rules. The dissection of animals as well as collection of the material was performed in the field, after culling.

## Supplementary Information

Supplementary Figure S1.

## Data Availability

The datasets used and/or analysed during the current study are available from the corresponding author on reasonable request.
