# Supplementary material for: The occurrence and molecular identification of Thelazia spp. in European bison (Bison bonasus) in the Bieszczady Mountains
Source: Sci Rep. 2022 Dec 29;12:22508. doi: 10.1038/s41598-022-27191-x (PMC9800370; doi:10.1038/s41598-022-27191-x)
Supplement: Supplementary file 1 — Supplementary Figure S1. [file 41598_2022_27191_MOESM1_ESM.pdf]

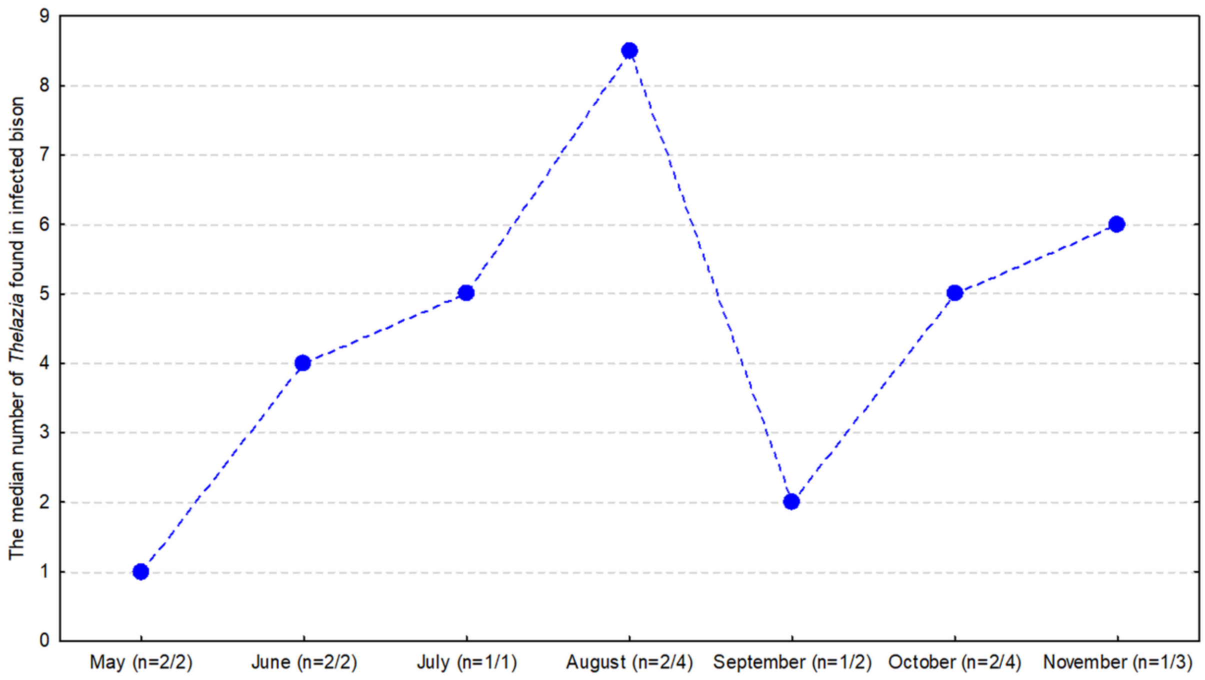

Supplementary figure S1. The median number of *Thelaziae* in an infected bison depending on the month of examination. The number of infected bison / the number of examined bison each month are presented in parentheses.
